# Realization of a Dental Framework by 3D Printing in Material Cobalt-Chromium with Superior Precision and Fitting Accuracy

**DOI:** 10.3390/ma13235390

**Published:** 2020-11-27

**Authors:** André Edelmann, Lisa Riedel, Ralf Hellmann

**Affiliations:** Applied Laser and Photonics Group, University of Applied Sciences Aschaffenburg, 63743 Aschaffenburg, Germany; s7040@h-ab.de (L.R.); ralf.hellmann@th-ab.de (R.H.)

**Keywords:** additive manufacturing, dental application, superior accuracy

## Abstract

We report on the generation of a cobalt-chromium dental framework with superior precision and fitting accuracy using selective laser melting. The objective of this study is the reduction of surface roughness and the possibility to manufacture a dental framework with high precision for passive fit with attachments, in particular a round tack. After selective laser melting, the dental framework is thermally post processed at 750 °C, shot-blasted with glass and highly polished. Nominal to actual 3D form deviation is analyzed by stripe light projection, revealing deviations being less than 250 μm, i.e., warpage is as low as to permit dental application and accurate passive fit. In particular, the critical area of the dental framework, the fixture to the implant (overdenture) shows negligible deviations. This superior fitting accuracy is confirmed by joining the bar with a testing stylus.

## 1. Introduction

Dental implant therapy is since more than 30 years a common practice in the contemporary restorative and surgical dentistry of partially and fully edentulous patients [1,2,3,4,5,6]. Different polymers and metals were qualified in terms of biocompatibility, with respect to their soft tissue responses and good clinical performance over long periods [7,8,9]. Among a variety of implant component shapes, one of the most distinguished representatives is the dental implant bar [10,11]. With reference to selective laser melting (SLM), as a beneficial additive manufacturing method for dental technology, titanium and cobalt-chrome alloys are the most prominent and approved metals for dental implant therapy [12,13,14,15].

**Figure 1:**
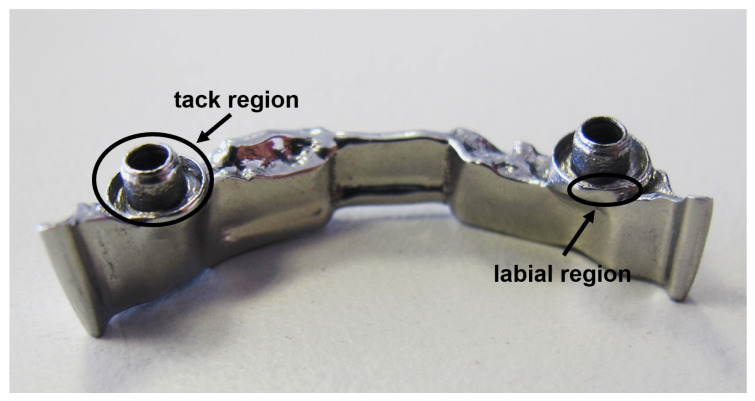
SLM fabricated dental framework.

For full anatomical restorations, the dental bar is designed to support partial dentures. Bars are typically employed in a variety of advanced designs with different, complex attachments and retentions (Figure 1 exemplifies details of such a bar), to take into account complex clinical situations and provide maximum comfort for the patient. Dental bars are manufactured in various geometries, such as, e.g., Preci-Horax bars, Dolder bars with joints, abutments and attachments, as well as freely designable bar shapes [10,16]. For a long time, cast bars with welded or soldered prefabricated attachments were the standard in implant-supported restoration of the maxilla and mandible [17,18]. However, these conventional restorations may shrink, expand or have unwanted inclusions as a result of the casting and joining processes [19,20]. During the last decade, precision milling evolved as current the state-of-the-art manufacturing technology to meet the increasing expectations in accuracy and stress-free fit as well as design flexibility [19,21,22].

Proper angulation and positioning of dental implants and the superstructure are essential to achieve acceptable prosthetic outcomes and long term success of implants and implant-supported prostheses, in turn striving superior demands on the fitting accuracy of bars with respect to shape divergence and surface roughness, e.g., in the labial region of the bar as indicated in Figure 1. In addition, surface properties should promote the integration of soft tissue while minimizing plaque and bacterial retention [23].

Therefore, different corrective techniques such as abrasive blasting, precision grinding or electrical discharge machining are employed to improve the surface and accuracy of dental bars. Though a clear definition of acceptable deviations in precision is not yet available [11], it was shown by Romero et al. and others that gaps in the range between 10 μm and 200 μm will be sufficient in meeting the criteria of passive fit between a cast bar superstructure and its interface with an implant abutment [24,25,26,27]. This prevents stress in the prosthesis-abutment-screw-implant complex, and, as a consequence, technical complications such as, e.g., fracture of the abutment, prosthesis or implant or biological complications including discomfort or pain to the patient or even bone fracture, respectively [9,28]. In fact, CNC-milled implant superstructures outperform the accuracy of fit that can be achieved with cast metal frameworks [29,30,31].

As a novel method in prosthodontics and orthodontics, additive manufacturing (AM) combines the advantages of high design flexibility, short manufacturing cycles enabling individual and small series production, high precision and a balanced selection of qualified polymer materials and metals. AM thus has the potential and is indeed already in use, beyond pure rapid prototyping, to replace conventional technologies, shorten process chains and improve dental implant technology [32,33,34]. Nonetheless, the demanding requirements in terms of precision and passive fit, as well as surface quality still challenge AM.

Against this background, we highlight the potential of SLM by exemplifying a complex shaped bar made of CoCrW. To facilitate the details of the complex design, in particular a thin walled hollow cylinder attachment as part of a full dental framework, which has not been previously reported by SLM, and to improve the surface roughness achievable with SLM, powders with different particle size distributions have been comparatively studied. The reduction of surface roughness is associated with reducing the necessary efforts in post processing, in turn enabling rapid, individualized manufacturing [11]. In addition and detail, achievable density and surface roughness have been optimized by investigating the influence of the applied laser fluence. Smallest shape deviations and high passive fit of highly polished bars lead to good passive fit as demonstrated by a testing stylus, typically used by dental laboratory technicians. In particular, the area of support of the tack that facilitates the connection to the implant is sensitive to roughness and shape deviations hampering precise passive fit.

## 2. Materials and Methods

### 2.1. Selective Laser Melting Conditions

For SLM, we employed a Realizer SLM 50 (Realizer GmbH, Bielefeld, Germany) equipped with a $P_{L}=100$ W yttrium fiber laser (IPG, YLM-100). Spot size of the laser is set to *d*_spot_ = 20 μm at focus position. The machine processes under argon atmosphere with a 0.1% oxygen level. The build platform was kept at $\vartheta_{\mathrm{plate}}=200$ °C to maintain the machined part at an elevated temperature as to avoid deformation by curling due to residual stress. The input energy density $E_{d}$ is varied in the range of 52 $J/{\mathrm{mm}}^{3}$ to 92 $J/{\mathrm{mm}}^{3}$ by varying the hatch distance $y_{\mathrm{hatch}}$ and keeping the laser power at *P*_L_ = 55 W, the exposure time at *t*_exp_ = 20 μm and the point distance at *x*_point_ = 6 μm, and layer thickness *z*_layer_ = 50 μm constant, respectively. The thus resulting energy density is calculated by Equation (1) [13,35].

(1) $$E_{d}=\frac{P_{L}\;t_{\exp}}{x_{\mathrm{point}}\;y_{\mathrm{hatch}}\;z_{\mathrm{layer}}}$$

### 2.2. Powder Characterization

In this study, we analyze and process commercially available CoCrW metal powder (Eisenbacher GmbH, Wörth am Main, Germany). The chemical composition of this powder is as listed in Table 1.

Important fabrication material parameters for processing metallic powder in AM systems are the flowability and the powder density. If the flowability is too low, a homogenous covering of powder over the build plate in the SLM machine cannot be ensured and, in addition, a low powder density can lead to a higher porosity of the fabricated components [36]. For a more detailed investigation of the build quality regarding the powder characteristics, we divide the powder by sieving into the fraction A32 and A45. The notation refers to the d90 value of the grain size distribution. The fraction in A32 contains powder where 90% of the grain sizes are below $d_{g}<32$ μm, whereas 90% of grain sizes in fraction A45 are below $d_{g}<45$ μm. For each of these two powder batches we measure the flow time and the powder density. The results are shown in Table 2. The flowability, which influences the coating of the powder bed, is determined by measuring the flow rate using a Hall-Flowmeter according DIN EN ISO 4490. The powder density, influencing the homogeneity and density of the powder bed and thus the accessible relative density of the SLM built part, is measured according DIN ISO 697.

Comparing powder sections A32 with A45, a higher flow time is found for A32. For this sieved particle size, the averaged grain sizes are smaller and, as a result, a higher probability of agglomeration by intermolecular interactions such as Van-der-Waals forces and higher contact surfaces of smaller particles come predominantly into play. As a result, higher stiction appears and the flowability is reduced [37,38]. In addition, the lowest powder density is found for A45, as for the A32 powder fraction an increase of finer grain sizes gets relevant and larger particles cannot fill the free spaces [39,40].

The moisture of the powder, which reduces the flow rate and thus the coating of the powder bed and which may also lead to higher gas porosity, is determined by weighting in combination with a drying chamber (PCE-MA 110, PCE Germany GmbH). We determine the moisture of both powder batches being under 0.01% and, therefore, exclude any influence of moisture to the flowability and powder density.

**Figure 2:**
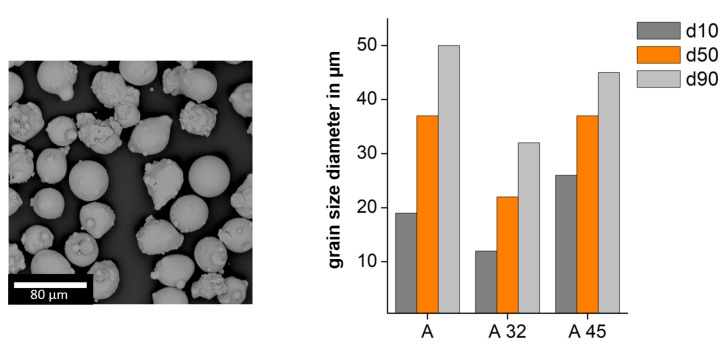
SEM images of Powder A (**left**) and particle size distribution of powder batch A, A32 and A45 (**right**).

Figure 2 depicts the morphology of the powder and the particle size distribution of the different powder batches A32 and A45. In the powder samples, a spherical characteristic with minor satellites is observed which are free of porosity. The particle size distribution of the powder was analyzed by a Camsizer X2 based on dynamic digital image analysis methods according to ISO 13322-2 (Retsch Technology GmbH). This particle size distribution and morphology allows good flowability and packing density, as was demonstrated in a flowability test conducted using the Hall flowmeter funnel.

### 2.3. Mechanical Properties

We evaluate the mechanical properties of SLM specimens based on powder batch A and comparing the results with the product specifications of CoCrW-powder (Eisenbacher Dentalwaren, Wörth am Main, Germany) [41]. By using tensile test specimens we measure the tensile strength with $\sigma_{m}=365$ MPa, Young’s modulus with E=170 GPa, elongation with $\epsilon_{b}=18\%$ and yield strength with $\sigma_{\mathrm{ys}}=567$ MPa ( AG-Xplus, Shimadzu, Kyoto, Japan). The Vickers hardness HV10/30 on the top surface of test cubes (edge length a=10 mm) results to $h_{\mathrm{vickers}}=365$ HV (Falcon 5000, Innovatest, Maastricht, The Netherlands). For the thermal expansion coefficient we obtain $\Delta l=14.5\times 10^{-6}\;K^{-1}$ (DIL 402 Expedis, Netzsch, Selb, Germany) by using cylindrical specimens with a diameter of d=6 mm and a length of l=50 mm and applying a heating rate of $r_{\mathrm{heat}}=5$ K/s up to $\vartheta_{\max}=300$ °C. The mechanical properties are combined in Table 3 and Table 4.

**Figure 3:**
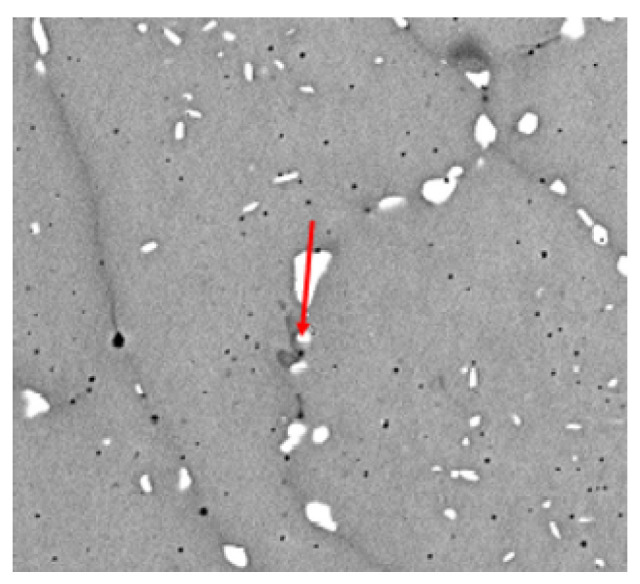
SEM image of CoCrW microstructure.

Additionally we have taken polished cut images of test specimens in combination with SEM and EDX analysis. We have found, in gerneral, homogeneous microstructure with grain boundaries, at which we observe segregations of Wolfram. In Figure 3 the images of the mircostructure is shown.

### 2.4. Experimental Flow Chart

**Figure 4:**
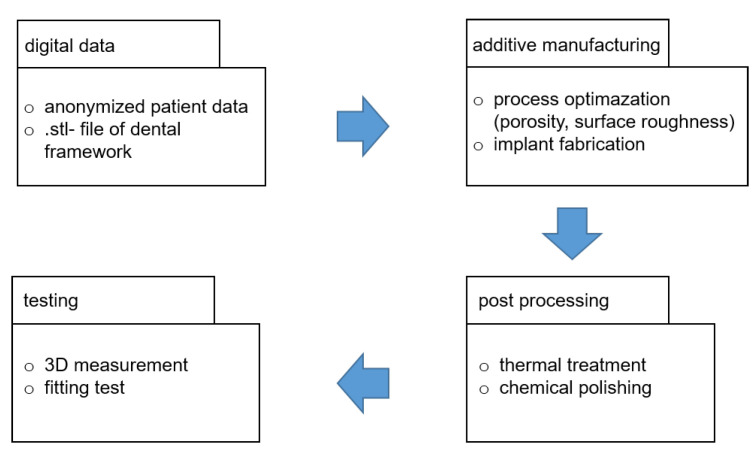
Experimental work flow carried out to fabricate the dental framework.

The flow chart of the experimental tasks carried out in this report is shown in Figure 4. Beside the metallurgical and physical examination of powders and test specimen, the generation of the dental framework is conducted as follows. Beginning from digital data, obtained by anonymized patient data and prepared in .stl-data format, we have additively built both specimen for testing and the dental bar by SLM (implant fabrication). Here we focus particularly on the surface roughness by optimizing the particle size distribution of the metal powder. After post processing by thermal treatment and polishing, the dental framework is characterized with particular focus on 3D form deviation and fitting accuracy.

## 3. Results and Discussion

### 3.1. Optimization of Density and Roughness

In medical application and in particular in dental restoration, a high quality of the additive manufactured parts is needed. An important quality factor refers to the remaining porosity in the SLM part. By using well defined process parameters, the porosity of SLM parts is, in general accessible, below one percent. As previous, comprehensive studies on selective laser melting have shown, the porosity in the material sensitively depends on the energy density [42,43,44,45,46]. If the energy density is too high, gas porosity appears and if, in contrast, the input of the energy is to low, lack-of-fusion porosity appears [47].

**Figure 5:**
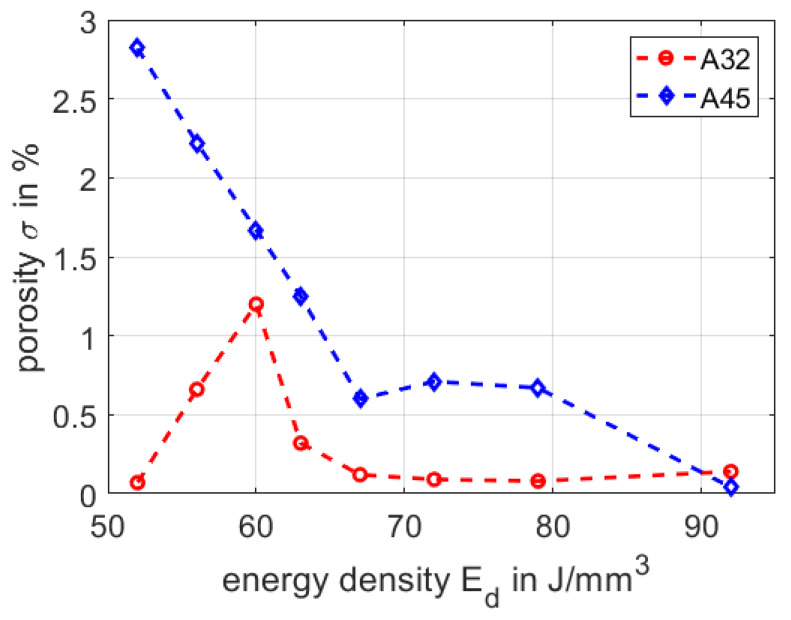
Comparison of the porosity of A32 and A45 in dependency of the energy density.

In the following, we compare the processing of the fine powder section A32 and the coarse powder A45, as to evaluate the remaining porosity in the volume of the SLM part. We process both powder batches with the same process parameters, yet varying the applied energy density from $E_{d}=52$ $J/{\mathrm{mm}}^{3}$ to $E_{d}=92$ $J/{\mathrm{mm}}^{3}$. While we keep the laser power $P_{L}$, the point distance $x_{\mathrm{point}}$, the exposure time $t_{\exp}$ and the layer thickness with $z_{\mathrm{layer}}$ constant, respectively, we vary the hatch distance $y_{\mathrm{hatch}}$ to accomplish different energy densities (as shown in Section 2.1). For each parameter set we built four cuboids with the dimensions of V=4×4×3 ${\mathrm{mm}}^{3}$ and measure the porosity by applying Archimedes method. The results for powder A32 and powder A45 are summarized in Figure 5.

By processing powder A45 and increasing the energy density, the porosity drops from σ=2.8% to σ=0.04%. We attribute this decrease to larger particle sizes, leading to lack-of-fusion porosities by lower energy inputs. In contrast, applying the finer powder A32, we find a peak value of the porosity with σ=1.2% at an energy density of $E_{d}=60$ $J/{\mathrm{mm}}^{3}$, whereas for energy densities between $E_{d}=67$ $J/{\mathrm{mm}}^{3}$ and $E_{d}=92$ $J/{\mathrm{mm}}^{3}$, respectively, we have measured a rather low and stable porosity between σ=0.1% and σ=0.2%. Here we assume the optimum between the lack-of-fusion and gas porosity. Considering both powder fraction A32 and A45, we find porosities of below one percent at higher energy densities of $E_{d}=67$ $J/{\mathrm{mm}}^{3}$. Therefore we define the process window for both powder fractions between $E_{d}=67$ $J/{\mathrm{mm}}^{3}$ and $E_{d}=92$ $J/{\mathrm{mm}}^{3}$. In this range the porosity is below one percent for both powder fractions, indicating an appropriate build quality.

One of the further limiting factors in the fabrication of high accuracy additive manufactured parts refers to the surface roughness, which we quantify by the common roughness parameter $R_{z}$. Deviations in the surface roughness lead to deviations in the accuracy of the part geometry and increase the necessary post processing efforts. Therefore, we discuss the two considered powder batches regarding the accessible surface roughness in dependence of the applied energy input during SLM. Surface roughness was analyzed using a laser scanning microscope (LSM VK-X200, Keyence).

**Figure 6:**
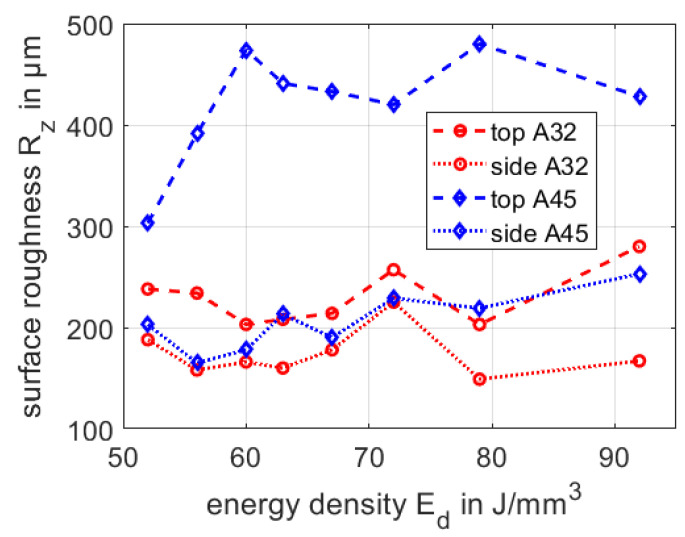
Comparison of surface roughness of the top and side surfaces.

The surface roughness of SLM parts, in general, depends on the grain size distribution of processed powder and used process parameters [48,49,50]. Furthermore, the surface roughness appears different on top and side surfaces, with respect to the build direction. In Figure 6 the surface roughness $R_{z}$ is shown for the top and side surfaces for the powder A32 and powder A45 as function of the applied energy density. A suitable process window regarding the porosity is already defined by the porosity between the energy density of $E_{d}=63$ $J/{\mathrm{mm}}^{3}$ and $E_{d}=92$ $J/{\mathrm{mm}}^{3}$.

Comparing the surface roughness as given in Figure 6, $R_{z}$ of the top surface of powder A45 is highest with $R_{z}>400$ μm, whereas the corresponding values of A32 (top), A32 (side) and A45 (side), respectively, are below $R_{z}<300$ μm. The roughness of the top surfaces of specimen made of A32 and A45 can jointly be explained by the particle size distribution. By processing coarser powder particles a rough surface appears due to sintering of particles on the top surface. The cavities between coarser powder particles cannot be filled with finer powder particles. In contrast, the side surfaces roughness of powder A45 and A32 is rather similar to each other, i.e., the influence of particle size distribution is less pronounced. Here smaller particles of both powder batches are sintered at the side surfaces and the quality of the SLM side surfaces depends mainly on the energy input [50]. As an interim conclusion, best surface roughness for the top and side surfaces is found for powder batch A32 with $R_{z}<300$ μm for the top and $R_{z}<200$ μm for the side. For dental application a low value of the surface roughness is needed allowing the fabrication of geometries with high accuracy and lowest effort in surface finishing of the respective components.

### 3.2. Fabrication of Dental Framework

**Figure 7:**
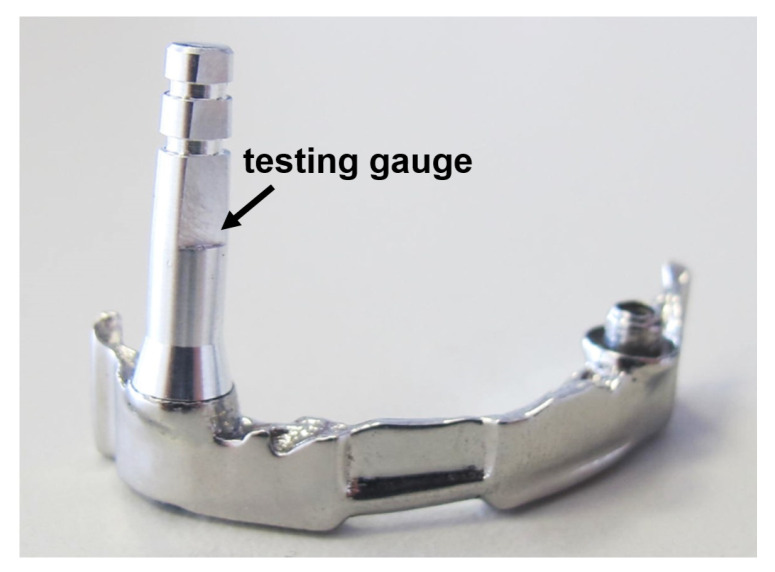
Dental framework with testing gauge.

To highlight the potential of of CoCrW for dental applications, we exemplify the SLM fabrication by generating a sophisticated dental framework (dental bar with attachements). In Figure 7 the finished dental framework is shown, after post processing with a thermal treatment of up to 750 °C, glass spheres blasting of the surface (grain size of 90–150 μm) and chemical polishing (the polishing step is done by a dental laboratory). Thermal treatment was carried out in a standard oven (Nabertherm) at a temperature of 750 °C (heating phase 45 min), temperature hold for 55 min and natural cooling. The resulting fitting accuracy is tested by a dental testing gauge (stylus) without significant deviation.

**Figure 8:**
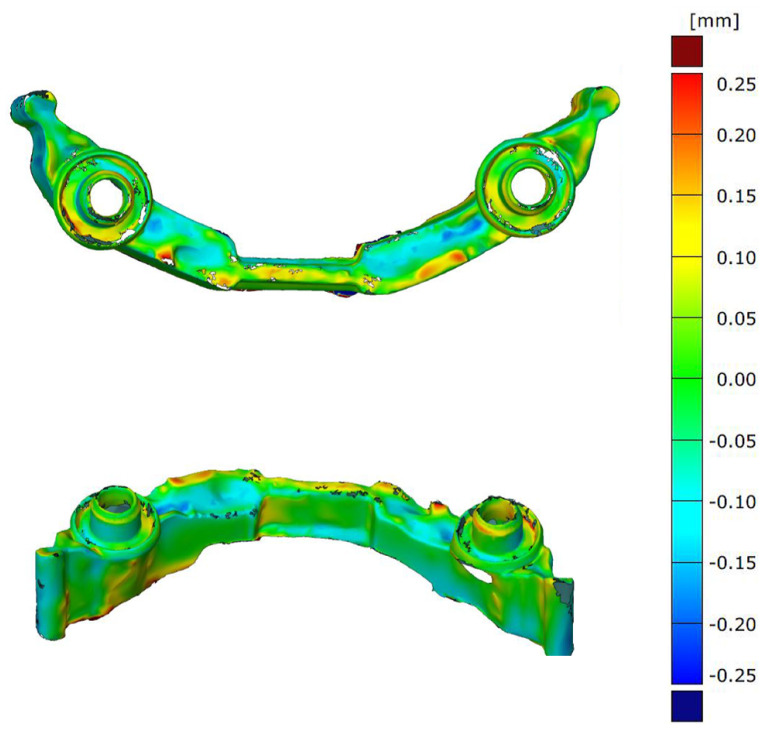
Nominal/actual value comparison of the dental framework—top view (above) and side view (below).

Verification of the geometrical accuracy of the dental framework is done by 3D inspection (3D scanning) and comparison of the actual values with the nominal values. Shape deviations and shape variance analysis as compared to the original CAD data is provided by stripe line projection using Atos Core and ATOS Professional V8-SR1 software (GOM, Brunswick, Germany). The results are shown in Figure 8 in different orientations. The maximum deviation for the whole dental framework is less than $d_{x}\leq 0.25$ mm. This fabrication accuracy excludes thermal warpage of the structure and well-defined energy input during the build process. In particular the critical area around the fixture of the dental implant shows very good precision with deviations to the nominal value of less than $d_{x}\leq 15$ μm.

## 4. Conclusions

We demonstrated additive manufacturing of a dental framework (dental bar) with superior precision and fitting accuracy. In particular, using a fine sized particle distribution leads to significant improvement of the surface roughness fulfilling the recommendations for dental applications. A proper setting of the SLM laser and scanning parameters, in particular the applied laser energy density, leads to a minimized shape deviation and warping of the fabricated dental implant. As a result and to exemplify and highlight the potential of selective laser melting, a CoCrW dental implant components, a dental bar with attachments on the contact area and small sized round tack with thin walls and sufficient roughness, is demonstrated, which is commonly difficult to process with conventional processes.

## Figures and Tables

**Table 1:** Chemical composition of CoCrW powder.

wt%	Cobalt	Chromium	Tungsten	Silicon	Manganous	Iron	Others
Powder	62.44	27.50	8.09	1.52	0.2	0.11	<0.1

**Table 2:** Flowability and powder density of powder A32 and A45.

	A 32	A45
flow time (s)	19.1 ± 0.1	14.5 ± 0.3
powder density (g/${\mathrm{cm}}^{3}$)	3.6 ± 0.1	3.1 ± 0.1

**Table 3:** Tensile strength, Young’s modulus, elongation and yield strength of SLM fabricated CoCrW specimens.

	Tensile Strength	Young’s Modulus	Elongation	Yield Strength
SLM	969 MPa	170 GPa	18%	567 MPa
reference [41]	1021 MPa	196 GPa	8%	642 MPa

**Table 4:** Vickers hardness and thermal expansion coefficient of SLM fabricated CoCrW specimens.

	Hardness	Thermal Expansion
SLM	365 HV	$14.5\times 10^{-6}\;K^{-1}$
reference [41]	356 HV	14.25 × $10^{-6}\;K^{-1}$

## Abbreviations

The following abbreviations are used in this manuscript:

SLM	selective laser melting
AM	additive manufacturing
